# When retraction replaces rebuttal: suppression of critical scholarship on parental alienation

**DOI:** 10.3389/frma.2026.1807122

**Published:** 2026-04-28

**Authors:** Keith Robert Head

**Affiliations:** Independent Researcher, Lubbock, TX, United States

**Keywords:** academic freedom, child custody, domestic violence, family court, academic suppression, parental alienation, retraction ethics, scientific suppression

## 1 Introduction

Parental alienation (PA) remains a deeply contested concept in psychiatry, psychology, law, and social work. While the occurrence of alienating behaviors is widely accepted, disagreement spans its validity as a construct, its use in custody adjudication and forensic assessment, and the interpretation of its empirical research base (Bernet, 2020; Harman et al., 2022; Meier, 2020; Kline Pruett et al., 2023). Originally introduced by Gardner (1985) as Parental Alienation Syndrome (PAS), the concept has been excluded from the DSM-5, with the vice-chair of the DSM-5 Task Force stating there was ‘not sufficient scientific evidence to warrant its inclusion' (Crary, 2010). The World Health Organization likewise removed it from the ICD-11, determining it ‘is not a health care term' (World Health Organization, 2020). Both declined to endorse the construct and pointed clinicians to broader categories of relational problems (Bernet et al., 2016; World Health Organization, 2020). The American Psychological Association (APA) Task Force on Violence and the Family, the National Council of Juvenile and Family Court Judges, and the American Professional Society on the Abuse of Children have either rejected the construct or cautioned against its forensic use (American Psychological Association, 1996; Dalton et al., 2006; AFCC and NCJFCJ, 2022; APSAC, 2023). The APA itself has stated that it holds no official position on ‘parental alienation syndrome' as a diagnostic construct (American Psychological Association, 2008), and its custody evaluation guidelines recognize ‘alienating behaviors' as a parenting pattern evaluators should be alert to, without endorsing parental alienation as a construct, syndrome, or diagnosis (American Psychological Association, 2022). The UN Special Rapporteur on Violence Against Women and Girls characterized it as a ‘discredited and unscientific pseudo-concept' (Alsalem, 2023). Proponents dispute these characterizations and have published research arguing that parental alienation is supported by a substantial and growing evidence base (Harman et al., 2022), while critics maintain the construct lacks empirical validation (Meier, 2020).

The concept continues to play a role in custody proceedings in multiple countries, making the terms of the debate consequential for courts, practitioners, and families. This opinion article does not revisit the full scientific case regarding PA, which has been investigated extensively elsewhere (Bruch, 2001; Meier, 2020; Milchman et al., 2020; Harman et al., 2022). Instead, it reviews a distinct and underexplored problem, namely the pattern in which proponents of PA have responded to critical scholarship through organized campaigns to retract, withdraw, or remove that work from the record rather than through the ordinary mechanisms of scientific debate. This pattern warrants attention because the integrity of scientific discourse depends on the principle that disagreement is met with evidence, not with coercion.

## 2 A documented pattern of suppression

The most extensively documented suppression effort targeted Challenging Parental Alienation: New Directions for Professionals and Parents by Mercer and Drew (2021), an edited volume published by Routledge. Following its publication, prominent PA proponents submitted a 126-page critique requesting retraction, endorsed by 45 organizations. When Routledge declined, noting the book had undergone external review and received supportive endorsements, the campaign escalated to the Committee on Publication Ethics (COPE). COPE contacted Routledge; the publisher again refused to withdraw the book. The proponents subsequently published a guest post on Retraction Watch framing their campaign as an effort to combat “misinformation” (Harman and Bernet, 2023). Disagreement with findings or interpretations is not grounds for retraction. COPE's retraction guidelines define retraction as a mechanism for correcting the literature when an article's flaws run deep enough that its ‘findings and conclusions cannot be relied upon,' citing major error, data or image irregularities, misrepresentation (e.g., fraud, identity theft, or fictitious authorship), unethical research, and compromised peer review. Where correction sufficiently resolves the concerns and the main results and conclusions stand, the guidelines identify correction, not retraction, as the remedy (COPE Council, 2025). None of these conditions appear to have applied.

Similar campaigns targeted multiple other publications. When Meier (2020) published her National Institute of Justice-funded empirical study of custody outcomes involving PA allegations, the National Parents Organization collaborated with the Parental Alienation Study Group (PASG) to formally request its retraction (Hubin, 2021). Global Action Research Integrity in Parental Alienation (GARI-PA) sought formal retraction of Vaccaro and Payueta's (2009) Spanish-language book El Pretendido Síndrome de Alienación Parental from publisher Desclée de Brouwer, producing a technical analysis recommending its immediate withdrawal (Amaro et al., 2023). GARI-PA also targeted Diesen and Diesen's, 2013 Swedish legal text published by Norstedts Juridik, escalating the effort to a misconduct complaint at Stockholm University, where Diesen held an emeritus position (Bernet, 2020). Bernet et al. (2015), all affiliated with the parental alienation research community, published a commentary in Children and Youth Services Review explicitly demanding the withdrawal of Clemente and Padilla-Racero's (2015a) article, which had questioned the empirical basis of Gardner's claims about parental manipulation. The journal declined to retract.

When UN Special Rapporteur Alsalem published her 2023 report recommending that states prohibit the use of PA in family law proceedings, PASG and GARI-PA published a 70-page denunciation and organized a campaign to prevent her from presenting the report to the Human Rights Council (Aichenbaum et al., 2023b). In response, the Rackman Center at Bar-Ilan University organized a Collective Expert Academic Response signed by an international consortium of scholars. That response documented reports from multiple countries of attempts by PA proponents to pressure academic institutions into disciplining or terminating faculty who question the concept, characterizing these efforts as ‘harassment campaigns aimed at suppressing and penalizing dissent' (The Ruth Emanuel Rackman Center, 2023). This pattern is consistent with Bernet and Xu's (2023) citation analysis published in Behavioral Sciences and the Law, in which they called for over 40 articles and books to be corrected or withdrawn. These campaigns have not been confined to a single country, with retraction demands and institutional complaints targeting critical scholarship in the United States, Spain, and Sweden, among others.

However, these suppression dynamics are not solely confined to high-profile publications. Following the publication of my own critical narrative review of the PA literature (Head, 2026), I received multiple emails from PA proponents pressuring me to retract or withdraw the article. One individual, prompted by the article, also contacted my academic institution in an apparent attempt to obtain personal information about me. At least one of these individuals failed to disclose their affiliations with PA proponent organizations when contacting me. No published rebuttal or competing review accompanied these communications. In most cases, the correspondence involved challenges to credibility, pushing theory, and persuasion rather than engaging with the empirical evidence itself. In my opinion, the responses did not meaningfully engage with the arguments but were an effort to secure retraction when the conversation failed to shift my position.

By contrast, a search of the published record, including Retraction Watch archives, COPE proceedings, documented publisher correspondence, and major article databases such as PubMed, PsycINFO, and Web of Science, failed to identify any instance in which critics of parental alienation have initiated formal retraction campaigns against publications by PA advocates. Critics have instead engaged through conventional academic channels, publishing rebuttals, counter-analyses, and empirical critiques in peer-reviewed journals, which remains the standard mechanism through which scientific disagreement is expected to operate (Meier et al., 2022; Mercer, 2021; Puppo, 2018). Retraction under COPE guidelines exists to correct the literature when flaws run deep enough that an article's findings can no longer be trusted, not to adjudicate disputes over interpretation or methodology (COPE Council, 2025).

However, none of the documented campaigns initiated by PA advocates have rested on research misconduct in the conventional sense. The complaints have instead centered on what proponents characterize as misinformation. The critique submitted to Routledge was titled A Comprehensive Review of Misinformation and Other Inaccuracies in Challenging Parental Alienation: New Directions for Professionals and Parents (Aichenbaum et al., 2023a), and the term recurs in the titles of the same proponents' own publications (Bernet, 2020; Bernet and Xu, 2023). In the research literature, misinformation denotes information considered incorrect based on ‘the best available evidence from relevant experts at the time' (Vraga and Bode, 2020, p. 138), or information ‘contrary to the epistemic consensus of the scientific community regarding a phenomenon' (Swire-Thompson and Lazer, 2020, p. 434). Where that evidence and expert opinion remain divided, as outlined above, the label treats a contested interpretation as settled by evidence or consensus. This framing conflates disagreement over theory and evidence with scientific misconduct and repurposes integrity mechanisms for what are substantive scholarly disputes. This pattern has been identified in other contested domains, where groups with professional or commercial interests in maintaining a concept's credibility have sought to suppress critical scholarship through institutional complaint processes rather than empirical rebuttal (Diethelm and McKee, 2009). The asymmetry documented here therefore arguably reflects a meaningful difference in how each side has approached scholarly disagreement. Critics of PA have predominantly engaged through publication and counter-analysis, while proponents have at times pursued administrative mechanisms that were not designed to resolve substantive scientific disputes.

## 3 Scientific dissent and retraction ethics

Not all organized retraction efforts are inappropriate. When multiple researchers identify fabrication, data manipulation, or undisclosed conflicts that compromise a study's integrity, collective action to seek retraction can be a necessary corrective. What distinguishes the campaigns documented here is that the objections center on interpretation and conclusions rather than on the forms of misconduct retraction guidelines were written to address. At least two of the cases documented here illustrate the distinction. The commentary demanding withdrawal of Clemente and Padilla-Racero's (2015a) study reviewed the study's own data and concluded that more than 40 percent of the children who witnessed no aggression had been manipulated to describe a non-event (Bernet et al., 2015). The original authors replied that their critics had simultaneously declared the data invalid and relied on those same data to support Gardner's theory (Clemente and Padilla-Racero, 2015b). A withdrawal demand resting on a contrary reading of the same data is a dispute over conclusions. The response to Meier (2020) followed the same course. Proponents formally requested retraction of the study from the George Washington law paper series that published it (Hubin, 2021) while simultaneously publishing a counter-study, based on an independently collected sample of appellate cases, asserting that its results disconfirmed Meier's findings (Harman and Lorandos, 2021), to which Meier and colleagues replied in detail (Meier et al., 2022). A counter-study disputes findings through the ordinary mechanism of scientific disagreement; a concurrent retraction request seeks removal of those findings from the record.

For their part, PA proponents would argue that their campaigns are not efforts at suppression but necessary interventions to protect the welfare of children and families. Bernet (2020) argued that misinformation about PA, once embedded in professional literature, is introduced into court through expert testimony and clinical guidance, leading practitioners to overlook PA when it occurs in the families they evaluate. In his view, allowing critical scholarship he considers inaccurate to remain uncorrected risks harming children because courts and clinicians may dismiss genuine cases of parental manipulation. Harman and Bernet (2023) extended this reasoning, stating that they believed the material in the book by Mercer and Drew (2021) was likely to damage children and families in multiple countries. Proponents frame their retraction requests not as attempts to silence dissent but as responsible efforts to prevent real-world harm. Critics, however, have argued that the danger runs in the opposite direction. They argue that the concept itself leads courts to dismiss genuine abuse, and what proponents label as misinformation is actually legitimate critique (Milchman et al., 2020; Meier, 2020). Both sides claim to be protecting children, but from opposite risks. What distinguishes these positions is not the concern for child welfare but the chosen mechanism of response, and whether retraction rather than rebuttal is the appropriate answer to scholarly disagreement. As the following section demonstrates, the broader literature on scientific suppression suggests otherwise.

The existing literature on scientific suppression provides important context. Martin (1999), a leading scholar on suppression of dissent in science, explains that when scientists produce work threatening powerful interests, predictable responses follow, including censorship, funding loss, and professional retaliation, typically framed as concerns about academic standards. He also showed that suppression and the appearance of consensus are functionally linked, since enforcing unanimity requires silencing dissent. This is echoed by Stevens et al. (2020), who analyzed the mechanisms through which what they call “denunciation mobs” suppress scholarship, arguing through recent case examples that coordinated collective pressure through petitions, social media, and complaint campaigns does not punish scholars directly but instead compels institutional authorities to do so, making those institutions complicit actors rather than neutral arbiters. For instance, a peer-reviewed paper on predatory publishing was retracted from Scientometrics after the chief executive of a major open access publisher complained directly to the journal and demanded retraction by a specific deadline. The editor complied, selecting hostile post-publication reviewers, ignoring the authors' counterarguments, and retracting without further deliberation. Every level of appeal, including COPE, which has a financial conflict of interest given its dependence on major publisher funding, failed to acknowledge any procedural failing (Srholec, 2024). Twenty-seven leading figures in the field, including members of the journal's own Distinguished Reviewers Board, publicly condemned the retraction as lacking justification, and the paper was subsequently republished in Quantitative Science Studies after independent editorial review (Abramo et al., 2022). The result here is a system in which research threatening established interests can be removed from the record through the same processes ostensibly designed to protect it.

The PA suppression pattern aligns with these frameworks. The campaigns do not identify fabrication or fraud. They target conclusions the campaigners dispute, and the remedy they pursue is removal from the literature. There is, of course, nothing objectionable about vigorous scientific disagreement. Science and research advance through critique, replication, and competing evidence. Proponents of PA are free to publish their own research, respond in commentaries, or produce empirical evidence addressing the validity concerns raised by critics. At times they have done so, for example Harman et al. (2022) published a systematic review in Developmental Psychology, and Bernet and Baker (2013) published a response to critics in the Journal of the American Academy of Psychiatry and the Law. These are legitimate forms of scientific engagement. The concern arises when the same proponents simultaneously pursue retraction campaigns, institutional complaints, and organized pressure on publishers to remove critical work. At that point, the activity operates outside scientific debate, functioning to narrow which conclusions remain available in the literature.

These dynamics are not unique to the parental alienation debate. In tobacco research, Philip Morris coordinated a sustained campaign against Stanton Glantz following his publications on secondhand smoke, deploying undisclosed industry-funded academics to attack his credibility, pressuring Congress to terminate his National Cancer Institute funding, and contacting his university to trigger institutional review, none of which engaged with his data (Landman and Glantz, 2009). In climate science, industry-affiliated organizations filed institutional complaints against Michael Mann after the IPCC published his findings, demanded his records through freedom of information requests, and publicly characterized his work as fraudulent. In every instance, he was fully cleared by the investigation (Mann, 2014). In both cases, the complaints operated outside formal publication channels where conflict-of-interest disclosure would have been required, the objections targeted researcher credibility rather than empirical content, and the campaigns functioned as deterrents regardless of outcome. To be clear, the comparison is one of mechanism rather than motive. PA proponents may be acting from genuine concern for children's welfare, but the structural similarity in how institutional complaint processes are used to bypass empirical engagement warrants examination regardless of the intent behind it. Lewandowsky et al. (2015) termed the downstream effect on the broader scientific community “seepage,” a process through which sustained external pressure causes researchers to self-moderate not in response to evidence but in anticipation of attack. The structural parallels with the PA retraction campaigns documented in this article suggest that what is occurring in the parental alienation field is not an anomaly but an instance of a recognized tactic that recurs wherever a body of research generates significant professional or institutional stakes.

## 4 Discussion

Two questions are often conflated in the PA debate that should be treated independently. The first is whether PA has scientific validity. The second is whether retraction campaigns constitute an appropriate response to scholarship questioning that validity. The argument presented in this article does not depend on resolving the first. Even a reader who accepts PA as a legitimate phenomenon should find the pattern documented here concerning, because the integrity of scientific discourse requires that disagreement be addressed through evidence rather than through administrative mechanisms designed for a different purpose. A concept with strong empirical foundations does not require the removal of its critics from the record; it survives scrutiny and is strengthened by it.

The practical stakes of this distinction are significant. Family courts increasingly ground custody decisions in peer-reviewed evidence. When critical analyses are retracted, chilled, or pressured out of the literature, the evidence base available to judges and evaluators is skewed toward one side of a debate in which genuine consensus does not exist. This does not serve children. It serves whichever party benefits from the appearance of consensus, and it compromises the quality of judicial decision-making in cases where children's safety is the central concern. The distortion is compounded by the fact that this skewing is largely invisible to the courts relying on the literature, since a retracted paper, though it remains in the database with a retraction notice, is effectively marked as discredited and excluded from standard citation practices, with no indication to the end user that the retraction resulted from organized lobbying rather than genuine scientific failing.

These stakes are compounded by financial interests that create conflicts of interest among many of those leading the retraction campaigns documented above. Reunification programs charge parents tens of thousands of dollars, with costs extending over months or years. Proponents also serve as paid expert witnesses, market proprietary diagnostic tools, and collect royalties on the books and manuals that form the evidentiary basis for alienation claims in court. The existence of these financial interests does not in itself prove that the retraction campaigns are financially motivated, and proponents may sincerely believe they are acting in the interest of children. Some proponents do disclose these interests in their formal journal publications (e.g., Bernet and Xu, 2023). It should also be noted that financial and professional interests are not exclusive to proponents of the concept. Critics of PA similarly serve as paid expert witnesses in custody proceedings and consult for legal teams representing parents in cases where alienation claims are raised. However, retraction campaigns, publisher complaints, COPE proceedings, and institutional misconduct complaints operate outside the structures where such disclosure is required, meaning that the editors, publishers, and institutional administrators receiving these demands have no formal requirements or mechanisms for evaluating whether complainants have material financial or other interests in the outcome. The same gap extends to pressure on individual researchers, consistent with the broader absence of disclosure norms outside formal publication channels. This structural gap matters because it is precisely in these informal channels that the suppression documented in this article takes place.

## 5 Limitations

This article has several limitations that should be considered. The cases documented here are drawn from publicly available records, published correspondence, and the author's own experience, not from a comprehensive empirical study of all retraction activity in the PA field. It is possible that suppression efforts by critics of PA exist but are not reflected in the public record, though the search described in this article did not identify any. It should also be noted that the author has previously published work critical of parental alienation, and that position informs how the cases in this article are presented and interpreted. Proponents would characterize their campaigns as responsible efforts to correct what they consider dangerous misinformation, and that framing reflects a sincerely held position even where the mechanism of response is, in the view of this author, misapplied. The asymmetry documented here should therefore be treated as a strong indication of a pattern rather than as definitive proof that suppression operates in only one direction.

## 6 Concluding thoughts

The pattern documented here points to a need for structural accountability in the retraction process. If organizations like COPE required third-party complainants to disclose financial interests and organizational affiliations when seeking retraction, the editors and administrators receiving those demands would at least have the information needed to evaluate them in context. Similarly, if journal editors evaluated organized retraction demands strictly against COPE's published criteria, the gap between what retraction is designed for and what it is being used for in this field would become harder to ignore. None of this requires silencing PA proponents. It requires the opposite. Let the debate proceed through the mechanisms science provides: peer-reviewed publication, empirical replication, and open scholarly exchange. The concern is not that proponents argue for their position but that they simultaneously pursue administrative channels not designed for that purpose in order to remove the opposing position from the record. Science advances through contestation, not through consensus enforced by editorial pressure. If critics are wrong, the evidence will show it. If they are right, the literature and the children it is meant to serve will be better for having heard them.
