# Correction: When retraction replaces rebuttal: suppression of critical scholarship on parental alienation

**DOI:** 10.3389/frma.2026.2004958

**Published:** 2026-10-14

**Authors:** Keith Robert Head

**Affiliations:** Independent Researcher, Lubbock, TX, United States

**Keywords:** academic freedom, child custody, domestic violence, family court, academic suppression, parental alienation, retraction ethics, scientific suppression

Following publication, external complaints regarding several statements in the article were raised through Frontiers' post-publication complaints process. After editorial review of those complaints and the revisions proposed by the author, the article has been corrected as described below.

Corrections have been made to **Introduction**, Paragraph 1.

This sentence previously read as: “Parental alienation (PA) remains one of the most contested concepts in psychiatry, psychology, law, and social work.”

The corrected passage appears below:

“Parental alienation (PA) remains a deeply contested concept in psychiatry, psychology, law, and social work. While the occurrence of alienating behaviors is widely accepted, disagreement spans its validity as a construct, its use in custody adjudication and forensic assessment, and the interpretation of its empirical research base (Bernet, 2020; Harman et al., 2022; Meier, 2020; Kline Pruett et al., 2023).”

This sentence previously read as: “Originally introduced by Gardner (1985) as Parental Alienation Syndrome (PAS), the concept has been excluded from the DSM-5 for insufficient evidence of validity and reliability and removed from the ICD-11 after the World Health Organization determined it ‘is not a health care term' (World Health Organization, 2020).”

The corrected passage appears below:

“Originally introduced by Gardner (1985) as Parental Alienation Syndrome (PAS), the concept has been excluded from the DSM-5, with the vice-chair of the DSM-5 Task Force stating there was ‘not sufficient scientific evidence to warrant its inclusion' (Crary, 2010). The World Health Organization likewise removed it from the ICD-11, determining it ‘is not a health care term' (World Health Organization, 2020). Both declined to endorse the construct and pointed clinicians to broader categories of relational problems (Bernet et al., 2016; World Health Organization, 2020).”

This sentence previously read as: “A substantial body of research disputes the validity of this concept as a syndrome, while recognizing that alienating behaviors can occur.”

This sentence has been removed.

This sentence previously read as: “The American Psychological Association, the National Council of Juvenile and Family Court Judges, and the American Professional Society on the Abuse of Children have either rejected or cautioned against its use, and the UN Special Rapporteur on Violence Against Women and Girls characterized it as a ‘discredited and unscientific pseudo-concept' (Alsalem, 2023).”

The corrected passage appears below:

“The American Psychological Association (APA) Task Force on Violence and the Family, the National Council of Juvenile and Family Court Judges, and the American Professional Society on the Abuse of Children have either rejected the construct or cautioned against its forensic use (American Psychological Association, 1996; Dalton et al., 2006; AFCC and NCJFCJ, 2022; APSAC, 2023). The APA itself has stated that it holds no official position on ‘parental alienation syndrome' as a diagnostic construct (American Psychological Association, 2008), and its custody evaluation guidelines recognize ‘alienating behaviors' as a parenting pattern evaluators should be alert to, without endorsing parental alienation as a construct, syndrome, or diagnosis (American Psychological Association, 2022). The UN Special Rapporteur on Violence Against Women and Girls characterized it as a ‘discredited and unscientific pseudo-concept' (Alsalem, 2023).”

This sentence previously read as: “Proponents dispute these characterizations and have published research arguing that parental alienation is supported by a substantial and growing evidence base (Harman et al., 2022).”

The corrected sentence appears below:

“Proponents dispute these characterizations and have published research arguing that parental alienation is supported by a substantial and growing evidence base (Harman et al., 2022), while critics maintain the construct lacks empirical validation (Meier, 2020).”

A correction has been made to **Introduction**, Paragraph 2.

This sentence previously read as: “This opinion article does not revisit the full scientific case regarding PA, which has been investigated extensively elsewhere (Bruch, 2001; Meier, 2020; Milchman et al., 2020; Harman et al., 2022; Head, 2026).”

The corrected sentence appears below:

“This opinion article does not revisit the full scientific case regarding PA, which has been investigated extensively elsewhere (Bruch, 2001; Meier, 2020; Milchman et al., 2020; Harman et al., 2022).”

A correction has been made to **A documented pattern of suppression**, Paragraph 1.

This sentence previously read as: “COPE's retraction guidelines specify that disagreement with findings or interpretations is not grounds for retraction; retraction is reserved for fabrication, falsification, plagiarism, unethical research, or compromised peer review (COPE Council, 2025).”

The corrected passage appears below:

“Disagreement with findings or interpretations is not grounds for retraction. COPE's retraction guidelines define retraction as a mechanism for correcting the literature when an article's flaws run deep enough that its ‘findings and conclusions cannot be relied upon,' citing major error, data or image irregularities, misrepresentation (e.g., fraud, identity theft, or fictitious authorship), unethical research, and compromised peer review. Where correction sufficiently resolves the concerns and the main results and conclusions stand, the guidelines identify correction, not retraction, as the remedy (COPE Council, 2025).”

A correction has been made to **A documented pattern of suppression**, Paragraph 2.

This sentence previously read as: “Bernet et al. (2015), all affiliated with the parental alienation research community, published a commentary in Children and Youth Services Review explicitly demanding the withdrawal of Clemente and Padilla-Racero's (2015) article, which had questioned the empirical basis of Gardner's claims about parental manipulation.”

The corrected sentence appears below:

“Bernet et al. (2015), all affiliated with the parental alienation research community, published a commentary in Children and Youth Services Review explicitly demanding the withdrawal of Clemente and Padilla-Racero's (2015a) article, which had questioned the empirical basis of Gardner's claims about parental manipulation.”

A correction has been made to **A documented pattern of suppression**, Paragraph 3.

This sentence previously read as: “That response documented reports from multiple countries of attempts by PA proponents to pressure academic institutions into disciplining or terminating faculty who question the concept, characterizing these efforts as ‘harassment campaigns aimed at suppressing and penalizing dissent' (The Ruth Emanuel Rackman Center, 2023).”

The corrected sentence appears below:

“That response documented reports from multiple countries of attempts by PA proponents to pressure academic institutions into disciplining or terminating faculty who question the concept, characterizing these efforts as ‘harassment campaigns aimed at suppressing and penalizing dissent' (The Ruth and Emanuel Rackman Center, 2023).”

Corrections have been made to **A documented pattern of suppression**, Paragraph 5.

This sentence previously read as: “Retraction exists under COPE guidelines to address fabrication, plagiarism, and serious ethical violations, not to adjudicate disputes over interpretation or methodology (COPE Council, 2025).”

The corrected sentence appears below:

“Retraction under COPE guidelines exists to correct the literature when flaws run deep enough that an article's findings can no longer be trusted, not to adjudicate disputes over interpretation or methodology (COPE Council, 2025).”

This passage previously read as: “However, none of the documented campaigns initiated by PA advocates have alleged misconduct in that conventional sense. The complaints have instead centered on what complainants characterize as misinformation, a framing that conflates theoretical disagreement with scientific misconduct and repurposes integrity mechanisms for what are substantive scholarly disputes.”

The corrected passage appears below:

“However, none of the documented campaigns initiated by PA advocates have rested on research misconduct in the conventional sense. The complaints have instead centered on what proponents characterize as misinformation. The critique submitted to Routledge was titled A Comprehensive Review of Misinformation and Other Inaccuracies in Challenging Parental Alienation: New Directions for Professionals and Parents (Aichenbaum et al., 2023a), and the term recurs in the titles of the same proponents' own publications (Bernet, 2020; Bernet and Xu, 2023). In the research literature, misinformation denotes information considered incorrect based on ‘the best available evidence from relevant experts at the time' (Vraga and Bode, 2020, p. 138), or information ‘contrary to the epistemic consensus of the scientific community regarding a phenomenon' (Swire-Thompson and Lazer, 2020, p. 434). Where that evidence and expert opinion remain divided, as outlined above, the label treats a contested interpretation as settled by evidence or consensus. This framing conflates disagreement over theory and evidence with scientific misconduct and repurposes integrity mechanisms for what are substantive scholarly disputes.”

A correction has been made to **Scientific dissent and retraction ethics**, Paragraph 1.

This sentence previously read as: “What distinguishes the campaigns documented here is that the objections center on interpretation and conclusions rather than on the forms of misconduct retraction guidelines were written to address.”

The corrected passage appears below:

“What distinguishes the campaigns documented here is that the objections center on interpretation and conclusions rather than on the forms of misconduct retraction guidelines were written to address. At least two of the cases documented here illustrate the distinction. The commentary demanding withdrawal of Clemente and Padilla-Racero's (2015a) study reviewed the study's own data and concluded that more than 40 percent of the children who witnessed no aggression had been manipulated to describe a non-event (Bernet et al., 2015). The original authors replied that their critics had simultaneously declared the data invalid and relied on those same data to support Gardner's theory (Clemente and Padilla-Racero, 2015b). A withdrawal demand resting on a contrary reading of the same data is a dispute over conclusions. The response to Meier (2020) followed the same course. Proponents formally requested retraction of the study from the George Washington law paper series that published it (Hubin, 2021) while simultaneously publishing a counter-study, based on an independently collected sample of appellate cases, asserting that its results disconfirmed Meier's findings (Harman and Lorandos, 2021), to which Meier and colleagues replied in detail (Meier et al., 2022). A counter-study disputes findings through the ordinary mechanism of scientific disagreement; a concurrent retraction request seeks removal of those findings from the record.”

Corrections have been made to **Scientific dissent and retraction ethics**, Paragraph 3.

This sentence previously read as: “They identify conclusions the campaigners oppose and seek to remove them from the literature.”

The corrected sentence appears below:

“They target conclusions the campaigners dispute, and the remedy they pursue is removal from the literature.”

This sentence previously read as: “At that point, the activity departs from scientific debate and becomes an effort to control the boundaries of permissible discourse.”

The corrected sentence appears below:

“At that point, the activity operates outside scientific debate, functioning to narrow which conclusions remain available in the literature.”

A correction has been made to **Concluding thoughts**, Paragraph 1.

This sentence previously read as: “Similarly, if journal editors evaluated organized retraction demands strictly against COPE's published criteria, which reserve retraction for fabrication, falsification, plagiarism, and serious ethical violations, the gap between what retraction is designed for and what it is being used for in this field would become harder to ignore.”

The corrected sentence appears below:

“Similarly, if journal editors evaluated organized retraction demands strictly against COPE's published criteria, the gap between what retraction is designed for and what it is being used for in this field would become harder to ignore.”

The following references have been corrected:

The reference for Bernet and Xu (2022) was written as

Bernet, W., and Xu, S. (2022). Scholarly rumors: citation analysis of vast misinformation regarding parental alienation theory. *Behav. Sci. Law* 41, 231–245. doi: 10.1002/bsl.2605

It now reads

Bernet, W., and Xu, S. (2023). Scholarly rumors: citation analysis of vast misinformation regarding parental alienation theory. *Behav. Sci. Law* 41, 231–245. doi: 10.1002/bsl.2605

The corresponding in-text citations in **A documented pattern of suppression**, Paragraph 3, and **Discussion**, Paragraph 3, now read Bernet and Xu (2023).

A second 2023 work by Aichenbaum and colleagues has been added to the reference list, so the existing entry, written as

Aichenbaum, Y. R., Brzosowski, B., Cedervall, B., Hellstern, B., Korosi, S., Ludmer, B., et al. (2023). *An Analysis of the Report by the Special Rapporteur on Violence Against Women and Girls*. Available online at: https://www.pasg.info/s/Analysis-Report-Special-Rapporteur-2023-06-02.pdf (Accessed February 08, 2026).

now carries the suffix b and reads

Aichenbaum, Y. R., Brzosowski, B., Cedervall, B., Hellstern, B., Korosi, S., Ludmer, B., et al. (2023b). *An Analysis of the Report by the Special Rapporteur on Violence Against Women and Girls*. Available online at: https://www.pasg.info/s/Analysis-Report-Special-Rapporteur-2023-06-02.pdf (Accessed February 08, 2026).

The corresponding in-text citation in **A documented pattern of suppression**, Paragraph 3, now reads Aichenbaum et al. (2023b).

A second 2015 work by the same authors has been added to the reference list, so the existing entry for Clemente and Padilla-Racero (2015), written as

Clemente, M., and Padilla-Racero, D. (2015). Are children susceptible to manipulation? The best interest of children and their testimony. *Child. Youth Serv. Rev*. 51, 101–107. doi: 10.1016/j.childyouth.2015.02.003

now carries the suffix a and reads

Clemente, M., and Padilla-Racero, D. (2015a). Are children susceptible to manipulation? The best interest of children and their testimony. *Child. Youth Serv. Rev*. 51, 101–107. doi: 10.1016/j.childyouth.2015.02.003

The corresponding in-text citation in **A documented pattern of suppression**, Paragraph 2, now reads Clemente and Padilla-Racero (2015a).

The article cited as Head (2026) has since been withdrawn by the publisher, so the entry, written as

Head, K. R. (2026). The scientific case against parental alienation: a critical review. *Integr. J. Res. Arts Human*. 6, 63–69. doi: 10.55544/ijrah.6.1.11

now reads

Head, K. R. (2026). The scientific case against parental alienation: a critical review. *Integr. J. Res. Arts Human*. 6, 63–69. doi: 10.55544/ijrah.6.1.11 [Withdrawn by publisher].

The following references have been added due to the corrections above:

AFCC and NCJFCJ (2022). *AFCC and NCJFCJ Joint Statement on Parent-Child Contact Problems*. Available online at: https://ncjfcj.org/wp-content/uploads/2022/08/NCJFCJ-AFCC-Joint-Statement.pdf (Accessed July 14, 2026).

Aichenbaum, Y. R., Bernet, W., Cedervall, B., Harman, J. J., Mendoza-Amaro, A., and Sherry, A. (2023a). *A Comprehensive Review of Misinformation and Other Inaccuracies in Challenging Parental Alienation: New Directions for Professionals and Parents*. Available online at: https://www.pasg.info/s/Analysis-of-Mercer-Drew-2023-04-04.pdf (Accessed July 14, 2026).

American Psychological Association (1996). *Violence and the Family: Report of the American Psychological Association Presidential Task Force on Violence and the Family*. Washington, DC: American Psychological Association. Available online at: https://eric.ed.gov/?id=ED399073 (Accessed July 14, 2026).

American Psychological Association (2008). *Statement on Parental Alienation Syndrome*. Available online at: https://www.apa.org/news/press/releases/2008/01/pas-syndrome (Accessed August 24, 2026).

American Psychological Association (2022). *Guidelines for Child Custody Evaluations in Family Law Proceedings*. Available online at: https://www.apa.org/about/policy/child-custody-evaluations.pdf (Accessed August 24, 2026).

APSAC (2023). *APSAC Announces Revisions to its Definitions of Psychological Maltreatment*. Available online at: https://apsac.org/apsac-announces-revisions-to-its-definitions-of-psychological-maltreatment/ (Accessed July 14, 2026).

Bernet, W., Wamboldt, M. Z., and Narrow, W. E. (2016). Child affected by parental relationship distress. *J. Am. Acad. Child Adolesc. Psychiatry* 55, 571–579. doi: 10.1016/j.jaac.2016.04.018

Clemente, M., and Padilla-Racero, D. (2015b). Facts speak louder than words: science versus the pseudoscience of PAS. *Child. Youth Serv. Rev*. 56, 177–184. doi: 10.1016/j.childyouth.2015.07.005

Crary, D. (2010). *Is Parental Alienation a Mental Disorder? CBS News*. Available online at: https://www.cbsnews.com/news/is-parental-alienation-a-mental-disorder/ (Accessed July 14, 2026).

Dalton, C., Drozd, L. M., and Wong, F. Q. F. (2006). *Navigating Custody and Visitation Evaluations in Cases With Domestic Violence: A Judge's Guide*. Reno, NV: NCJFCJ. Available online at: https://www.ncjfcj.org/wp-content/uploads/2012/02/navigating_cust.pdf (Accessed July 14, 2026).

Harman, J. J., and Lorandos, D. (2021). Allegations of family violence in court: how parental alienation affects judicial outcomes. *Psychol. Public Policy Law* 27, 184–208. doi: 10.1037/law0000301

Kline Pruett, M., Johnston, J. R., Saini, M., Sullivan, M., and Salem, P. (2023). The use of parental alienation constructs by family justice system professionals: a survey of belief systems and practice implications. *Fam. Court Rev*. 61, 372–394. doi: 10.1111/fcre.12716

Swire-Thompson, B., and Lazer, D. (2020). Public health and online misinformation: challenges and recommendations. *Annu. Rev. Public Health* 41, 433–451. doi: 10.1146/annurev-publhealth-040119-094127

Vraga, E. K., and Bode, L. (2020). Defining misinformation and understanding its bounded nature: using expertise and evidence for describing misinformation. *Polit. Commun*. 37, 136–144. doi: 10.1080/10584609.2020.1716500

The author states that these corrections do not change the scientific conclusions of the article in any way.

The original version of this article has been updated.

